# Amyloid precursor protein as a fibrosis marker in infants with biliary atresia

**DOI:** 10.1038/s41390-024-03582-w

**Published:** 2024-09-28

**Authors:** Jan C. Kamp, Omid Madadi-Sanjani, Marie Uecker, Christopher Werlein, Lavinia Neubert, Joachim F. Kübler, Mikal Obed, Norman Junge, Tobias Welte, Jannik Ruwisch, Danny D. Jonigk, Jan Stolk, Gertrud Vieten, Sabina Janciauskiene

**Affiliations:** 1 Department of Respiratory and Infectious Medicine, Hannover Medical School, Hannover, Germany; 2 Biomedical Research in Endstage and Obstructive Lung Disease Hannover (BREATH), German Center for Lung Research (DZL), Hannover, Germany; 3 Centre of Pediatric Surgery, Hannover Medical School, Hannover, Germany; 4 Institute of Pathology, Hannover Medical School, Hannover, Germany; 5 Division for Pediatric Gastroenterology and Hepatology, Department of Pediatric Kidney, Liver and Metabolic Diseases, Hannover Medical School, Hannover, Germany; 6 Institute of Pathology, RWTH Aachen University Medical Faculty, Aachen, Germany; 7 Department of Pulmonology, Leiden University Medical Center, Member of European Reference Network Lung, Section Alpha-1-Antitrypsin Deficiency, Leiden, The Netherlands

## Abstract

**Background:**

Biliary atresia (BA) is a rare condition of unknown origin in newborns with jaundice. In BA bile ducts are non-functional, causing neonatal cholestasis and following liver fibrosis and failure.

**Methods:**

This retrospective study included liver biopsies of 14 infants with BA aged [mean ± SD] 63 ± 23 days. Patients were grouped according to the clinical course (jaundice-free vs recurrent jaundice vs required liver transplantation or liver fibrosis (Ishak fibrosis score)) and followed for 1.61–5.64 years (mean 4.03). Transcriptome profiles were assessed using a panel of 768 fibrosis-specific genes, reanalyzed via qRT-PCR, and confirmed via immunostaining. Plasma from an additional 30 BA infants and 10 age-matched controls were used for amyloid precursor protein (APP) quantification by ELISA.

**Results:**

Different clinical outcome groups showed a homogeneous mRNA expression. Altered amyloid-metabolism-related gene expression was found between cases with Ishak fibrosis score greater than 4. Immunostaining confirmed a distinct presence of APP in the livers of all BA subjects. APP plasma levels were higher in BA than in age-matched controls and correlated with the histological fibrosis grade.

**Conclusions:**

These results suggest that amyloidosis may contribute to BA and liver fibrosis, indicating that APP could serve as a potential liquid biomarker for these conditions.

**Impact:**

- Biliary atresia patients with higher fibrosis scores according to Ishak have higher hepatic expression of amyloid-related genes while amyloid precursor protein accumulates in the liver and increases in the circulation.
- After a recent study revealed beta-amyloid deposition as a mechanism potentially involved in biliary atresia, we were able to correlate amyloid-metabolism-related transcript levels as well as amyloid precursor protein tissue and plasma levels with the degree of hepatic fibrosis.
- These findings suggest that amyloid precursor protein is a fibrosis marker in infants with biliary atresia, reinforcing the role of amyloid metabolism in the pathogenesis of this serious disease.

## Introduction

Biliary atresia (BA) is a liver disease of the newborn that untreated leads to progressive liver fibrosis and end-stage cirrhosis and even with treatment, the necessity of liver transplantation during childhood is likely. BA is a rare disease affecting about 1:15,000 newborns worldwide annually. BA is characterized by the obstruction of bile ducts and progressive fibrotic tissue remodeling within the first months of life. Data on the etiology and pathogenesis of the disease as well as the identification of markers that can predict the severity and progression of the disease are still missing and are currently the subject of clinical research. Infants with BA have been described as having a common clinical presentation with some variability and several associated extrahepatic manifestations such as gallbladder anomalies, bile duct cysts, cytomegalovirus infection, immune-related destruction of the biliary system, preterm birth, and/or laterality defects.^1^ In addition, it has been reported that infants of non-Hispanic black mothers are more likely to develop BA than those of non-Hispanic white mothers suggesting a genetic background of the disease.^2,3^

The main feature of the disease is obstruction of bile flow from the liver due to atretic extrahepatic bile ducts. Removing this obstruction is the focus of the Kasai surgery, also known as hepatoportoenterostomy (KPE), a procedure developed in the 1950s to connect the liver and small intestine to produce intestinal bile secretion.^4,5^ Regardless of the postoperative results, liver transplantation is ultimately required in most cases due to progressive liver fibrosis.^6^

Several investigations reported distinct genes as potential risk factors for BA in specific populations. For instance, genome-wide association studies (GWAS) performed in Caucasian populations identified 3 different candidate gene loci as predisposing factors: the 2q37.3 locus containing the glypican 1 (*GPC1*) gene,^7^ a susceptibility locus at the 14q21.3 region containing the ADP-ribosylation factor 6 (*ARF6*),^8^ and a susceptibility locus in the 2p16.1 region containing the EGF-containing fibulin extracellular matrix protein 1 (*EFEMP1*).^9^ Another GWAS performed in a Chinese population identified a susceptibility locus at the 10q24.2 region containing 2 genes: adducin 3 (*ADD3*), which is involved in cytoskeleton assembly, and X-prolyl aminopeptidase 1 (*XPNPEP1*), which is involved in metabolism and inflammation.^10^ In addition, a recent report suggested *FOXA2*, *CFC1*, *ZEB2*, *ZIC3*, *HNF1B*, and *PKD1L1* as well as *STIP1* and *REV1* as candidate susceptibility genes of BA.^11^ A recent study, published by Glessner J et al.^12^ *revealed* a novel association between BA requiring liver transplantation and the top-ranked *AFAP1* (Actin Filament Associated Protein 1) gene, as well as the second-ranked *TUSC3* (Tumor suppressor candidate 3) gene. This groundbreaking discovery emerged from the largest GWAS of BA cases and genetically matched controls. Additionally, research by Luo et al.^13^ uncovered a distinct 14-gene signature within the liver affected by BA, accurately predicting the likelihood of survival with the native liver at 2 years of age. These genes appear to be involved in various biological processes, including immune responses, inflammation, fibrosis, and bile duct development. While each gene’s specific function may vary, collectively, they contribute to the pathogenesis and progression of BA. In this study, we applied targeted transcriptome profiling in retrospectively collected liver biopsies of 14 BA infants to evaluate the fibrosis-related gene expression at the time of KPE. Our aim was to gain additional insights into the elusive pathophysiology of this rare and severe disease.

## Materials and methods

### Patients

The retrospective cohort of BA infants was collected between 2016 and 2020 in accordance with the guidelines of the Ethical Committee of the Hannover Medical School (approval # 41/2000, # 8601_BO_K_2019, # 9270_BO_K_2020). 14 infants from 37 to 110 days of age with confirmed BA by radiological, cholangiographical, and laboratory examination, without alternative causes of neonatal cholestasis such as neonatal hepatitis, Alagille’s syndrome, congenital liver fibrosis, choledochal cyst, cystic fibrosis, α-1 antitrypsin deficiency, or metabolic abnormalities were excluded. Out of all infants, 13 (93%) underwent KPE and 1 received a liver transplantation (LiverTx) as a primary therapy. The patients were subgrouped according to clinical characteristics: first, infants who did not require LiverTx after KPE but subsequently developed recurrent jaundice were referred to as the natural liver survival group (SNL, *n* = 3) and compared with infants who survived jaundice-free with natural liver (JF-SNL, *n* = 3) as well as to infants who subsequently underwent LiverTx. The LiverTx group was further divided into subjects with early LiverTx within one year after KPE and subjects with LiverTx after the first year post-surgery (LTx_ET and LTx_LT, *n* = 4, respectively). Second, infants with severe liver fibrosis based on results of liver biopsies performed during KPE or LiverTx (Ishak fibrosis score >4, *n* = 6) were compared to those with less advanced liver fibrosis (Ishak fibrosis score <5, *n* = 8). The postoperative observation period was 4.03 years (range 1.61–5.64 years). During KPE, liver biopsies were taken from the peripheral liver surface of segment VI for consecutive histopathological analysis and scored for the extent of fibrosis analog to the Ishak fibrosis score from 0 (no fibrosis) to 6 (cirrhosis).^14^

An additional cohort of 30 BA infants and 10 age-matched controls (liver-healthy infants with inguinal hernia) with available plasma samples were used for plasma protein analysis by enzyme-linked immunoadsorbent assay (ELISA). Clinical characteristics are summarized in Table 1 and more detailed clinical characteristics as well as data on sample utilization are presented in Supplementary Table S1.

**Table 1:** Clinical characteristics.

	BA tissue samples	BA plasma samples		Control plasma samples
Sample size (*n*)	14	33	Sample size	10
Female Sex (%)	50	57	Female sex	10
Age (days)	63 ± 23	52 ± 20	Age	61 ± 21
Weight at KPE (g)	4736 ± 799	4514 ± 799	Weight at surgery	3993 ± 726
Clinical outcome (*n*)				
JF_SNL	3	8		
SNL	3	6		
LTx_ET	4	11		
LTx_LT	4	0		
Death	0	1		
n/a	0	4		
Syndromic variant (*n*)	3	4		
Ishak fibrosis score				
2	2	1		
3	3	6		
4	3	8		
5	6	4		

*BA* bile atresia, *KPE* Kasai portoenterostomy, *JF_SNL* jaundice-free survival with native liver, *SNL* survival with native liver, *LTx_ET* early liver transplantation ≤1 year after KPE, *LTx_LT* late liver transplantation >1 year after KPE, *n/a* not available.

### Institutional perioperative standard of care and follow-up

All patients were treated with ursodeoxycholic acid and oral fat-soluble vitamins prior to surgery. In case of increased international normalized ratio (INR) at admission, Vitamin K was given intravenously and, if needed, frozen fresh plasma was transfused. Postoperatively, additional intravenous antibiotic therapy with a 3rd generation cephalosporine was given for a minimum of seven days. Afterward, patients were switched to oral cholangitis prophylaxis with cotrimoxazole for 3–6 months. Patients were given rectal corticosteroid (Budesonide) from the 3rd postoperative day for three months. MCT-enriched milk was added to the diet in variable portions depending on weight gain and availability of breast milk. Patients were followed up regularly in the outpatient gastroenterological clinic. Follow-up exams included ultrasound and laboratory analysis as well as clinical evaluation. In the case of cholangitis, patients were treated with intravenous antibiotics for at least 4 weeks. In the case of progressive cholestasis, progressive liver failure, and/or decompensated portal hypertension, patients were evaluated and listed for LiverTx.

### Transcriptome profiling

Histological analysis was performed by an experienced liver pathologist. Liver biopsies were obtained during surgery, immediately snap-frozen in liquid nitrogen (still in the operation room), and stored at −80 °C. RNA isolation from frozen liver biopsy and quality control was performed by Canopy Biosciences, St. Louis, MO, using their standard protocols (https://canopybiosciences.com/sample-guidelines-nanostring-services). Samples were analyzed using a commercial panel on 760 fibrosis-specific genes (nCounter Human Fibrosis V2 Panel, Nanostring Technologies, Seattle, WA) complemented by 8 bile acid-related genes as described elsewhere.^15^ Samples were analyzed using the probe-based nCounter analysis system (NanoString Technologies, Seattle, WA). Normalization of counts was performed using the nSolver analysis software version 4.0 (NanoString Technologies, Seattle, WA) and 10 internal reference genes. Measurements were standardized using established housekeeping genes (glucuronidase beta [GUSB] and phosphoglycerate kinase 1 [PGK1]). In-depth analysis of the differential gene expression was performed using R software version 3.2.2 (R Foundation for Statistical Computing, Vienna, Austria) and the nCounter Advanced Analysis module version 1.1.5 (NanoString Technologies, Seattle, WA). GraphPad Prism V9.0 (GraphPad Software, Boston, MA) was used for statistical comparisons.

### Immunohistochemistry and amyloid staining

Two µm-thick sections of paraffin-embedded formalin-fixed (FFPE) liver specimens from all 14 patients were used for immunostaining. After 2 × 10-min deparaffinization with xylene, decreasing ethanol concentrations were used for rehydration followed by heat-induced epitope retrieval in antibody buffer. Amyloid precursor protein (APP) was selected as the primary antibody target based on the mRNA expression results. Staining was performed according to the manufacturer’s protocol using the ZytoChem Plus HRP Polymer Kit (Zytomed Systems, Berlin, Germany) and 3,3’-diaminobenzidine solution. Eukitt mounting medium (Sigma Aldrich, Rockville, MD) was used as an adhesive and sealant. In addition, amyloid protein staining with Congo red was performed as previously described.^16^ Slide scanning was performed using the APERIO CS2 scanner and the ImageScope software version 12.3.3.5048 (both provided by Leica Biosystems, Wetzlar, Germany). Details about all used antibodies and dyes are shown in Table 2.

**Table 2:** Antibodies and dyes used for immunostaining and amyloid staining.

Target of antibody	Product name	Host species	Pretreatment	Dilution	Manufacturer
**APP**	Recombinant anti-amyloid precursor protein antibody [ab32136]	Rabbit	Tris buffer, pH 9.0	1:500	Abcam
**Amyloid protein**	**Congo red (CAS 573-58-0)**	n/a	80% ethanol with 0.001 M NaOH and 7% NaCl	3% saturated congo red in pretreatment buffer	Serva

*APP* amyloid precursor protein, *n/a* not applicable.

### Immunofluorescence microscopy

Clear sections were stained for amyloid precursor protein (clone Y188, ab32136, Abcam, Cambridge, United Kingdom). Briefly, clear sections were deparaffinized with xylol and ethanol and pretreated in TRIS (PH 9) for 30 min and 98 °C for antigen-retrieval followed by 10 min blocking in 10% donkey serum. The primary antibody was incubated at room temperature for one hour in a dilution of 1:500 followed by one washing step in washing solution (LOT Zuc020-2500, Zytomed Systems GmbH, Berlin, Germany). For fluorescence imaging, APP was visualized via a fluorescent donkey anti-rabbit secondary antibody (Alexa Fluor® 555 Donkey Anti-Rabbit IgG H&L, ab150062, Abcam, Cambridge, United Kingdom) followed by two washing steps in washing solution and deionized water for 5 min and mounting with DAPI/Dura-Tect-Solution ultra (LOT MT-0008-0.8, Zytomed Systems GmbH, Berlin, Germany). Images were taken on an Olympus APX100 microscope (Olympus, Tokyo, Japan) mounted with a Hamamatsu ORCA-Fusion camera (Hamamatsu Photonics, Hamamatsu, Japan). Image processing was carried out in ImageJ software^17^ and figures were generated in GIMP v2.10.

### Plasma protein quantification

Plasma APP protein was determined using a quantitative sandwich ELISA kit according to the producer’s recommendations (MyBioSource, Inc., CA, Catalog No: MBS451848). The assay reaction was stopped by the addition of sulphuric acid solution and the color change was measured spectrophotometrically at a wavelength of 450 nm using Microplate reader Tecan Infinite M200 (Männedorf, Switzerland). APP concentration in the samples was determined by comparing the O.D. of the samples to the standard curve. Protein detection Range 78.1–5000 pg/mL; sensitivity <35 pg/mL. Quality control assays assessing reproducibility identified the intra-assay CV (CV < 10%) and inter-assay CV (CV < 12%).

### Statistical analysis

Normality was tested for all analyses using the D’Agostino & Pearson test. Differential mRNA expression between 4 outcome groups was tested using the two-way ANOVA and correction for multiple testing was performed using Tukey’s multiple comparisons test while statistical comparison between the two Ishak fibrosis score groups was performed using unpaired t-tests with Welch correction complemented by correction for multiple comparisons via the Benjamini–Yekutiely method. Differential APP relative expression between Ishak fibrosis score groups assessed by PCR was analyzed using the ordinary one-way ANOVA. Analysis of differences in APP plasma levels between Ishak fibrosis score groups was conducted using the ordinary one-way ANOVA and comparisons between BA and control plasma levels were performed using unpaired t-tests. Differential protein expression between groups was tested using multiple Mann-Whitney U-tests. Statistical significance was categorized as *(*p* < 0.05), **(*p* < 0.01), ***(*p* < 0.001), and ****(*p* < 0.0001).

## Results

### Patient demographics

Fourteen BA infants (mean age 63 ± 23 days, 50% female) from Hannover Medical School were included in this study based on the availability of clinical data and liver biopsies. Of these, 8 had subsequently undergone LiverTx (4 within the first postoperative year and 4 after more than one-year post-KPE) and 6 survived with their own liver until the end of follow-up (3 without jaundice and 3 with recurrent jaundice). According to the Ishak fibrosis score, 6 of 14 patients showed advanced fibrosis (Ishak fibrosis score >4). The main indications for LiverTx were cholangitis with deterioration of liver function, progressive cholestasis, portal hypertension, and recurrent gastrointestinal bleeding. In addition, plasma samples from 30 BA subjects (mean age 52 ± 20 days, 57% female) and 10 age-matched controls (mean age 61 ± 21 days, 10% female) were included for further analysis.

### Targeted liver gene profiling

**Fig. 1:**
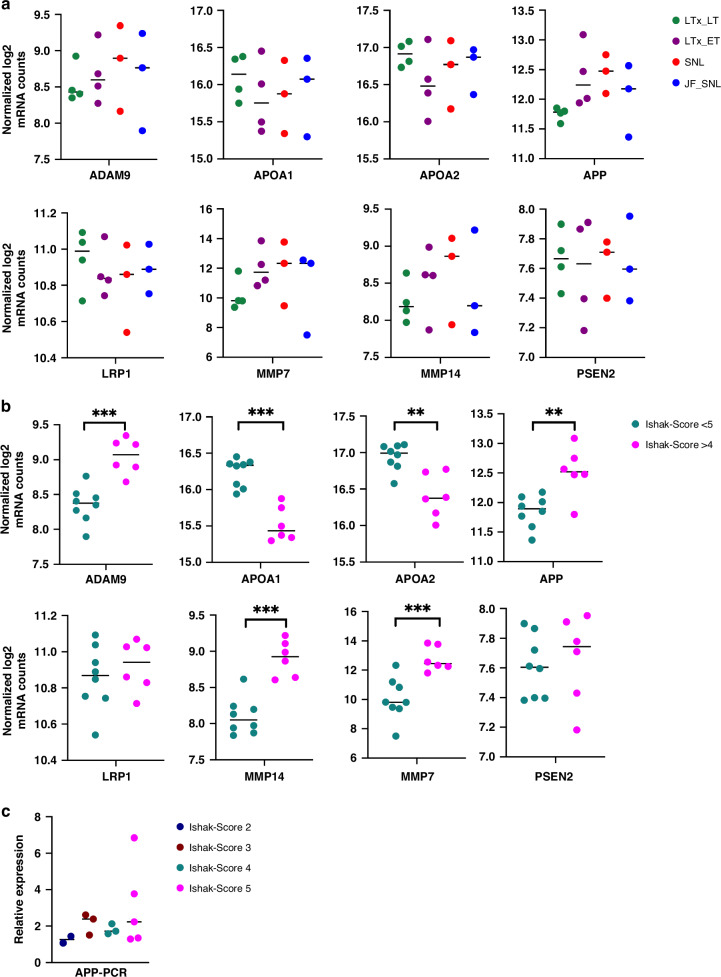
Amyloidosis-related gene expression. Dot plots showing normalized log2 mRNA counts of amyloid-metabolism-related genes in 4 different outcome groups (**a**) and in subjects with severe (Ishak fibrosis score >4) vs. less severe (Ishak fibrosis score <5) liver fibrosis (**b**). Additionally, APP-levels are compared between different Ishak fibrosis scores (**c**). LTx_LT, late liver transplantation (≥1 year); LTx_ET, early liver transplantation (<1 year); SNL, survival with the native liver; JF_SNL, jaundice-free survival with the native liver. Statistical significance was categorized as *(*p* < 0.05), **(*p* < 0.01), ***(*p* < 0.001), and ****(*p* < 0.0001).

We analyzed liver biopsies using a commercial panel of 760 fibrosis-specific genes (Fibrosis V2, Nanostring, Seattle, WA), supplemented by eight bile acid-related genes. Gene expression patterns did not differ between different clinical outcomes (jaundice-free vs recurrent jaundice vs required LiverTx). The principal component analysis (PCA) showed excellent clustering of groups with different fibrosis levels, while different outcome groups did not exhibit perceptible clustering, aligning with our gene expression results (Supplementary Fig. 1). However, compared to healthy liver tissues, elevated expression of APP was detected. We, therefore, focused on genes associated with amyloidosis (*ADAM9*, *APOA1*, *APOA2*, *APP*, *LPR1*, *MMP7*, *MMP14*, and *PSEN2*) that also showed no significant difference between the 4 groups (Fig. 1a*)*. Finally, all BA patients were re-examined histologically for degree of liver fibrosis and the gene expression analysis was repeated using the Ishak fibrosis score for group comparison instead of clinical outcomes. In this way, significant differences between cases with Ishak fibrosis score greater than 4 and less than 5 could be detected for the majority of genes related to amyloid metabolism (Fig. 1b). APP was selected as the most promising BA marker, analyzed in all BA tissue samples via qRT-PCR, and correlated with the respective Ishak scores. Tendentially, increasing APP levels were related to increasing Ishak fibrosis scores (Fig. 1c). Notably, with increasing Ishak fibrosis, well-known fibrotic markers in BA, such as COL1A1 and MMP2, showed expression patterns comparable to APP levels, whereas ACTA2 levels were less consistent (Supplementary Fig. 2).

### Qualitative and quantitative analysis of APP

**Fig. 2:**
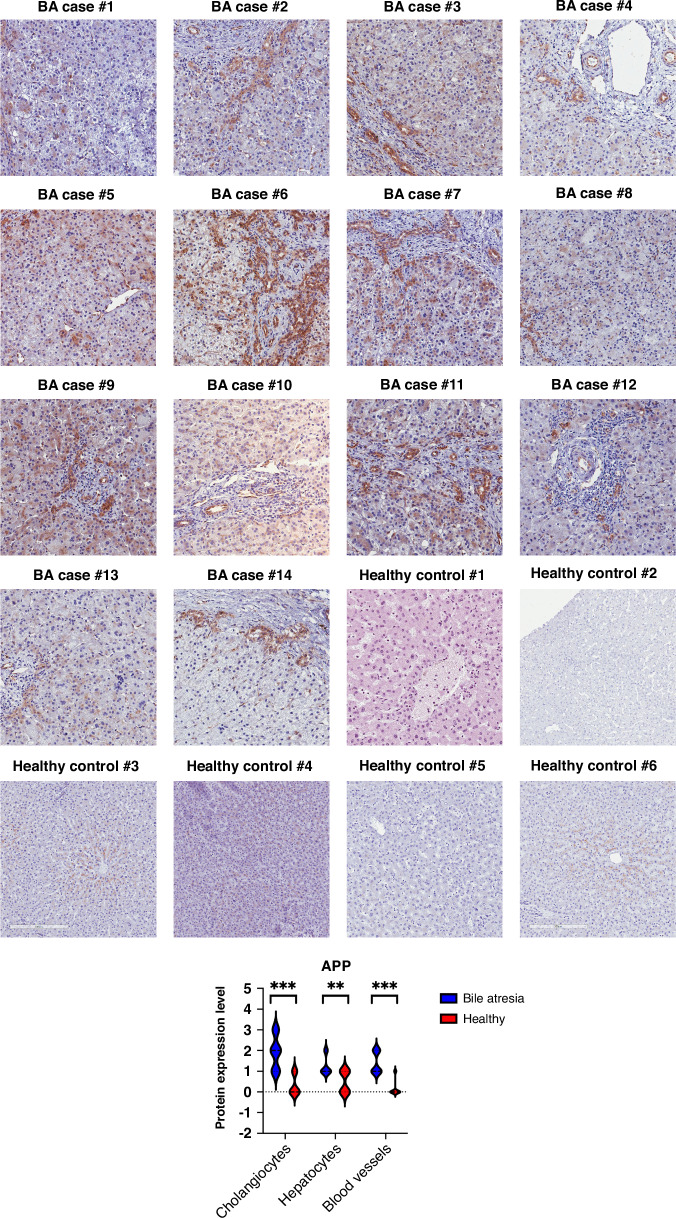
Immunostaining of liver APP performed using rabbit monoclonal antibody to APP. Bound antibodies were visualized using HRP-DAB (3,3′-diaminobenzidine tetrahydrochloride) staining. APP-positive areas are specifically stained in brown. Images were taken after automated whole-slide imaging using the APERIO CS2 scanner (Leica Biosystems, Wetzlar, Germany) and ImageScope software version 12.3.3.5048 (Leica Biosystems). Healthy liver tissue was used as a control. BA bile atresia. APP amyloid precursor protein. Statistical significance was categorized as *(*p* < 0.05), **(*p* < 0.01), ***(*p* < 0.001), and ****(*p* < 0.0001).

**Fig. 3:**
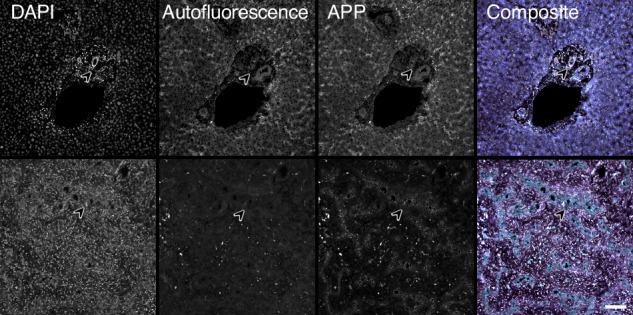
Representative immunofluorescence imaging of APP in control and biliary atresia liver. Figure showing immunofluorescence imaging of control liver (upper row) and bile atresia liver (lower row) of amyloid precursor protein (APP). Note the expression of APP in small intrahepatic bile ducts in bile atresia (arrowhead lower row), while there was no detectable expression of APP in intrahepatic bile ducts of the control liver (arrowhead upper row). APP was visualized using an Alexa Fluor 555 fluorescent secondary antibody. Autofluorescence imaging was generated using a GFP filter cube. Nuclei were stained with DAPI. Magnification 200×, scale bar equals 100 µm.

To learn more about the distribution of APP in the livers of BA infants, the protein was stained (using an antibody that recognizes amino acid residues 750 aa to the C-terminus of APP) in histological sections of BA livers and healthy controls. As shown in Fig. 2, specific staining for APP protein was found in all BA livers, mainly in the biliary epithelial cells or bile duct lumen. Occasionally, APP was detected in periportal hepatocytes, whereas no APP was detected in healthy control liver tissue. The specificity of APP stainings in bile atresia vs. healthy control liver tissue was verified via complementary immunostaining as demonstrated in Fig. 3. Due to the limited availability of patient material, we did not conduct detailed investigations on the extracellular or intracellular localization of APP. It is known that APP is primarily located in the endoplasmic reticulum and Golgi apparatus,^18^ and exploring this further warrants a separate study.

**Fig. 4:**
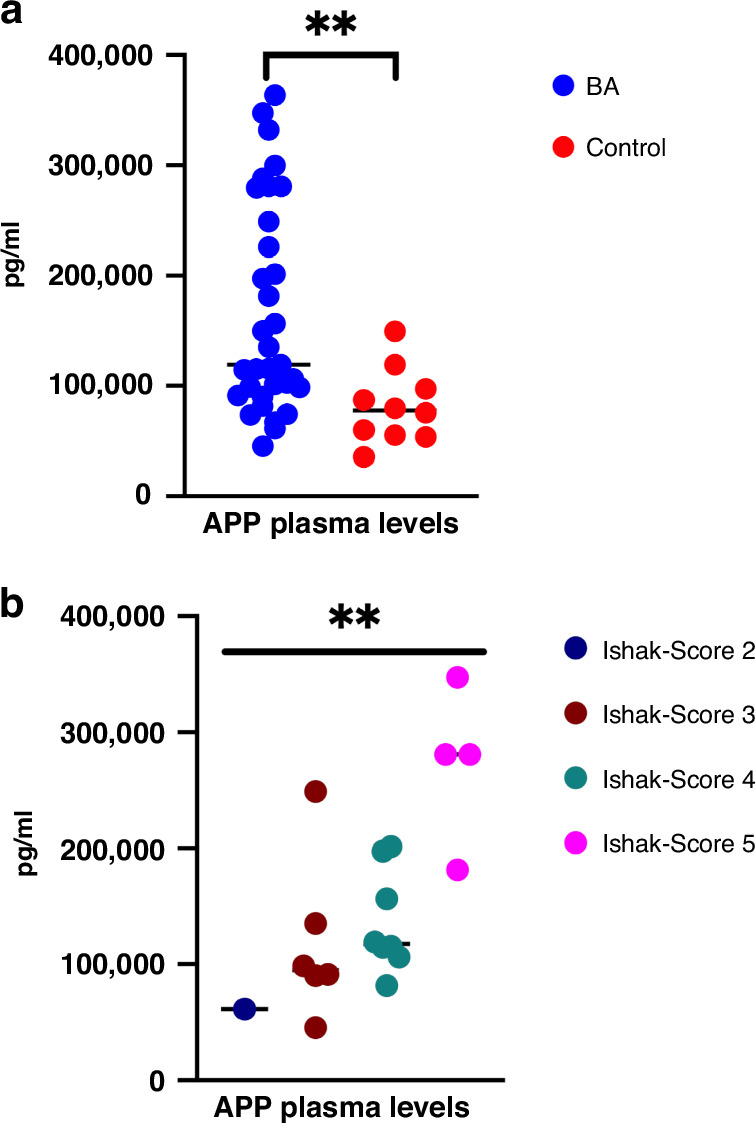
Plasma quantification of APP. **a** Dot plots showing plasma levels of APP in bile atresia (BA) subjects vs. age-matched children with inguinal hernia (control). **b** Dot plots showing APP plasma levels in subjects with different fibrosis severity assessed by Ishak fibrosis score. Data presented as mean (SD), and *P* values ≤ 0.05 were considered statistically significant. Statistical significance was categorized as *(*p* < 0.05), **(*p* < 0.01), ***(*p* < 0.001), and ****(*p* < 0.0001).

Regarding plasma levels, we found significantly higher APP protein levels in BA infants compared to age-matched controls without BA (Fig. 4a). Furthermore, the separation of BA cases based on Ishak fibrosis score showed a clear trend for the direct association between higher Ishak fibrosis scores and higher APP plasma levels (Fig. 4b).

### Comparison of three example BA cases used for all analyses

**Fig. 5:**
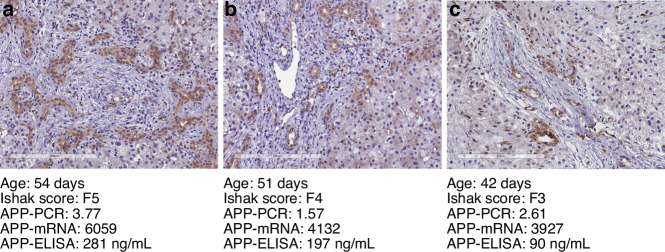
APP expression in exemplary subjects. Comparison of three exemplary BA cases (**a**–**c**) that were available for all analyses. Immunostaining of liver APP performed using rabbit monoclonal antibody to APP. Bound antibodies were visualized using HRP-DAB (3,3′-diaminobenzidine tetrahydrochloride) staining. APP-positive areas within hepatocytes are brown in color. BA bile atresia, APP amyloid precursor protein, PCR polymerase chain reaction, mRNA messenger ribonucleic acid, ELISA enzyme-linked immunosorbent assay.

The data described above suggests that APP is altered in BA and is related to Ishak’s fibrosis score. Of all 14 BA infant samples analyzed, we had three female cases with available corresponding liver tissue and plasma samples that were evaluated using all methods of our study (Fig. 5). Indeed, hepatic APP expression and plasma APP levels decreased in association with Ishak fibrosis scores 5, 4, and 3, respectively.

## Discussion

To date, the pathophysiology of BA remains largely elusive. Although KPE can effectively clear jaundice in many cases, liver fibrosis often persists and progresses to cirrhosis, necessitating LiverTx. One of the challenges in managing BA is the inability to accurately predict which infants will experience long-term benefits from KPE and which will eventually require LiverTx. Published data indicate that the need for LiverTx after KPE can range from 45% to 70% within the first 5 years following the procedure.^19^ This variability underscores the importance of better understanding the underlying mechanisms driving disease progression in BA, as well as identifying prognostic markers that can reliably predict patient outcomes and guide treatment decisions.

Several factors affect the outcomes of BA following KPE, including age at surgery, immune mediators, and the extent of liver fibrosis at the time of surgery.^20–23^ Other studies have suggested that the activation of hepatic stellate cells drives liver fibrosis post-KPE.^24,25^ Few studies have evaluated GGT as a marker of BA disease progression. The GGT-to-AST and ALT ratios were also studied.^26^ In our BA infant cohort, we did not find any significant differences in fibrosis-related gene expression in relation to liver enzyme levels between those who underwent KPE alone and those who later required, and without LiverTx. A high expression of collagen type 1 alpha 1 (COL1A1) and alpha-smooth muscle actin (α-SMA) genes in liver tissue at the time of KPE has been found to be associated with activation of myofibroblasts, a higher risk of LiverTx or death.^27–29^ High expression of transforming growth factor-beta 1 (TGF-β1), a cytokine involved in fibrosis, in liver tissue at the time of KPE was also associated with a higher risk of LiverTx or death.^30^ Cilia has many functions within the developing liver including mechano-, chemo- and osmo-sensoring. In response to changes in bile flow or composition, cilia can trigger signaling pathways that stimulate fluid secretion or absorption from the cholangiocytes. This control of bile flow through the cilia-mediated sensing mechanisms is essential for maintaining liver homeostasis.^31^ Different studies implicated defective ciliary structure and/or function as one of the mechanisms of fibrotic liver diseases (e.g., by a loss of cilia on cholangiocytes indicated by a loss of PKHD1 [the polycystin family proteins]).^32,33^ Functional analyses further demonstrated the absence of cilia in BA livers with *KIF3B* mutations, and knockdown of *KIF3B* and other genes in human control fibroblasts and cholangiocytes resulted in the reduced number of cilia.^34^ Human ciliopathies are also associated with a loss of Hedgehog pathway components, such as PTCH1 receptors, during embryonic development.^35^ The miRNA-21/PTEN/AKT axis promotes the fibrosis process in BA suggesting that ALK1 might be a potential therapeutic target.^36^ In our data set from the fibrosis gene panel, six cilia-related genes did not differ among BA infants after subgrouping according to clinical outcomes or fibrosis scores.

Different studies provide evidence that changes in fibrosis-related gene expression may be a useful predictor of BA progression and may help identify children who are at high risk of requiring LiverTx. In our study, 14 BA infants who received KPE (*n* = 13) or LiverTx (*n* = 1) as primary treatment did not show differences in their fibrosis-related gene expression between patients requiring transplantation after KPE and those with or without recurring jaundice after KPE.

A recent study by Babu et al. suggested an association between the accumulation of beta-amyloid around the bile ducts and bile duct pathology in BA. In addition, the authors found altered expression of several genes involved in amyloid-β precursor protein (APP) processing and the Alzheimer’s secretase signaling pathway.^37^ Other authors found amyloid-β expression increased in the plasma and livers of infants with BA^38^ and based on the liver APP gene expression suggested the utility of APP as a biomarker in conjunction with other liver function parameters for BA and non-BA cholestatic liver diseases.^39^

Therefore, of all the genes included in our fibrosis panel, APP and genes related to APP regulation and amyloidosis appeared to be the most interesting. Based on the previous publication by Babu et al., we found in our panel the following genes related to amyloidosis: *ADAM9, APOA1, APOA2, APP, LPR1, MMP7, MMP14*, and *PSEN2*. The hepatic transcript levels of these genes did not differ between subgroups with different clinical outcomes. However, the comparison of infants with advanced and less advanced fibrosis scores (Ishak fibrosis score >4 vs. <5) showed an increased expression of 4 amyloid-related genes (*ADAM9, APP, MMP7*, and *MMP14*) and a decreased expression of 2 amyloid-related genes *(APOA1, APOA2)* in infants with more advanced liver fibrosis.

A disintegrin and metalloprotease 9 (ADAM9) is a metalloprotease with various functions, which are mediated either by its disintegrin domain for adhesion or by its metalloprotease domain for the shedding of a large range of cell surface proteins.^40,41^ Among other functions, ADAM9 is involved in the regulation of APP cleavage.^42,43^ Apolipoproteins (ApoA1) and (ApoA2) are constituents of HDL and, according to experimental studies, are implicated in hereditary amyloidosis,^44^ and inflammation.^45^ Many studies have implicated the potential involvement of matrix metalloproteinases (MMPs), including MMP7 and MMP14, in the development and progression of fibrosis but also amyloidotic pathologies.^46^ Matrilysin (MMP7) was associated with tissue remodeling during the progression of liver fibrosis in BA, and serum MMP7 levels seem to help BA diagnosis.^47–49^ MMP14, also called MT1-MMP, is a type I transmembrane protein that can cleave gelatin, fibronectin, and laminin. Increased expression and activation of MMP14 occur during liver fibrogenesis.^50^ The MMP14-mediated signaling in fetal liver progenitor cells appears to promote biliary luminal formation around the portal vein and negatively control hepatocyte maturation.^51^

Based on the high expression of *APP* in BA liver biopsies with advanced fibrosis, we hypothesize that *APP* may be a driver of fibrogenesis offering potential as a predictive biomarker for progressive liver fibrosis. Higher levels of APP expression have been reported in BA compared to other causes of liver disease and control groups.^52^ Our immunostainings confirmed the high expression of APP at the protein level in the liver of BA infants, and ELISA-based plasma APP protein quantification showed higher APP in infants with more severe versus less severe liver fibrosis scores. However, in contrast to the data of Babu et al.,^37^ we did not find amyloid accumulation in BA infant livers via congo red stain and therefore were not able to confirm the presence of biliary amyloidosis. However, the antibody used to stain amyloid by Babu et al. recognizes both APP as well as amyloid-β, so it seems possible that the authors observed APP-positive cells but not amyloid per se. Further studies are needed to clarify this issue.

Identifying children with BA who are at high risk of disease progression and liver transplantation is crucial for timely intervention and optimal management of this disease. APP and related amyloidosis markers may help guide clinical decision-making and improve outcomes for children with BA.

It is important to point out that a recent study found a novel association of BA requiring LiverTx with the top-ranked *AFAP1* and second-ranked *TUSC3* genes in this largest-ever GWAS of BA cases.^12^ The *AFAP1* gene, also known as AFAP1L1, encodes a protein involved in actin cytoskeleton organization and cell motility. Its relationship with the APP gene is unknown although one can predict that AFAP1 may modulate the processing and trafficking of APP, potentially influencing the generation and aggregation of Aβ peptides.^53^ The *TUSC3* gene, also known as N33, has been associated with cell proliferation, apoptosis, and tumor suppression. Some studies suggest potential interactions or cross-regulations between *TUSC3* and *APP* gene pathways.^54,55^ In BA, liver injury and fibrosis are key pathological features, therefore it is plausible that TUSC3 and APP pathways may intersect through common signaling cascades related to inflammation, fibrosis, or cellular stress responses. For example, TUSC3 may modulate cellular processes involved in liver injury and fibrosis, potentially influencing the expression or processing of APP and its downstream effects on Aβ peptide production. Conversely, APP processing and Aβ peptide accumulation may impact cellular pathways regulated by TUSC3, potentially exacerbating liver injury and fibrosis in BA patients. Further research is warranted to elucidate the specific molecular mechanisms underlying the potential cross-regulations between TUSC3 and APP gene pathways in BA patients. A previous study reported that mRNAs encoding proteins that regulate fibrosis genes were increased in liver tissues from BA infants that did not survive for 2 years whereas mRNAs that encoded proteins that regulate glutathione metabolism were increased in BA infants that survived for 2 years.^13^ In line, the authors reported that the antioxidant N-acetyl-cysteine reduces liver injury and fibrosis in mice with BA. Hence, agents promoting glutathione metabolism might be important in BA treatment.

Taken together, our study was not able to detect molecular differences between BA infants with different clinical outcomes but provides evidence for amyloidosis-related genes as potential contributors to BA pathogenesis and subsequent liver fibrosis, where APP could serve as a potential liquid biomarker. Future studies with larger cohorts are needed to substantiate these results.

### Limitations

Our study is limited by the small number of included BA subjects, reducing the power of the analyses performed on subgroups, which makes drawing definitive conclusions difficult. In addition, no control group of non-BA neonatal cholestasis was available and the cohorts used for each assay in this study differed in parts due to limited sample availability.

Based on the routinely used amyloid staining methods, including Congo Red, we were not able to detect amyloid in newborn BA livers. Consequently, we did not pursue further analyses on Aβ peptides in the liver and plasma, which are the downstream products of APP. We believe that Aβ peptides are not generated in the first few months after birth and that their tissue deposition requires months to years as known from other adult diseases such as Alzheimer’s disease. These peptides might emerge later in life and this could be investigated in other cohorts with older patients.

## Supplementary information

Supplementary Figure S1

Supplementary Figure S2

Supplementary Table S1

## Data Availability

The datasets generated during and/or analyzed during the current study are available from the corresponding author on reasonable request.

## References

[1] Jiang, J. et al. Epidemiological characteristics and risk factors of biliary atresia: a case-control study. *BMJ Open.* **11**, e049354 (2021). 34903536 10.1136/bmjopen-2021-049354 PMC8671910

[2] Livesey, E. et al. Epidemiology of biliary atresia in England and Wales (1999–2006). *Arch. Dis. Child Fetal Neonatal Ed.* **94**, F451–F455 (2009). 19457876 10.1136/adc.2009.159780

[3] The, N. S. et al. National Birth Defects Prevention Study. Risk factors for isolated biliary atresia, National Birth Defects Prevention Study, 1997–2002. *Am. J. Med. Genet. A.* **143A**, 2274–2284 (2007). 17726689 10.1002/ajmg.a.31926

[4] Medappil, N. et al. Kasai portoenterostomy for biliary atresia - surgical precautions for better outcomes. *J. Pediatr. Surg.* **54**, 868–869 (2019). 30580834 10.1016/j.jpedsurg.2018.09.028

[5] Davenport, M. et al. Surgical and medical aspects of the initial treatment of biliary atresia: position paper. *J. Clin. Med.* **11**, 6601 (2022). 36362829 10.3390/jcm11216601 PMC9656543

[6] Madadi-Sanjani, O. et al. Long-term outcome and necessity of liver transplantation in infants with biliary atresia are independent of cytokine milieu in native liver and serum. *Cytokine* **111**, 382–388 (2018). 30300856 10.1016/j.cyto.2018.09.010

[7] Leyva-Vega, M. et al. Genomic alterations in biliary atresia suggest region of potential disease susceptibility in 2q37.3. *Am. J. Med. Genet A.* **152A**, 886–895 (2010). 20358598 10.1002/ajmg.a.33332 PMC2914625

[8] Ningappa, M. et al. The role of ARF6 in biliary atresia. *PLoS ONE* **10**, e0138381 (2015). 26379158 10.1371/journal.pone.0138381 PMC4574480

[9] Chen, Y. et al. A genome-wide association study identifies a susceptibility locus for biliary atresia on 2p16.1 within the gene EFEMP1. *PLoS Genet.* **14**, e1007532 (2018). 30102696 10.1371/journal.pgen.1007532 PMC6107291

[10] Garcia-Barceló, M. M. et al. Genome-wide association study identifies a susceptibility locus for biliary atresia on 10q24.2. *Hum. Mol. Genet.* **19**, 2917–2925 (2010). 20460270 10.1093/hmg/ddq196 PMC2893814

[11] Rajagopalan, R. et al. Exome sequencing in individuals with isolated biliary atresia. *Sci. Rep.* **10**, 2709 (2020). 32066793 10.1038/s41598-020-59379-4 PMC7026070

[12] Glessner, J. T. et al. Biliary atresia is associated with polygenic susceptibility in ciliogenesis and planar polarity effector genes. *J. Hepatol.* **79**, 1385–1395 (2023). 37572794 10.1016/j.jhep.2023.07.039 PMC10729795

[13] Luo, Z., Shivakumar, P., Mourya, R., Gutta, S. & Bezerra, J. A. Gene expression signatures associated with survival times of pediatric patients with biliary atresia identify potential therapeutic agents. *Gastroenterology* **157**, 1138–1152.e14 (2019). 31228442 10.1053/j.gastro.2019.06.017 PMC6756963

[14] Everhart, J. E. et al. Prognostic value of Ishak fibrosis stage: findings from the hepatitis C antiviral long-term treatment against cirrhosis trial. *Hepatology* **51**, 585–594 (2010). 20101752 10.1002/hep.23315 PMC3814134

[15] Kamp, J. C. et al. Fibrosis-related gene profiling in liver biopsies of PiZZ α1-antitrypsin children with different clinical courses. *Int J. Mol. Sci.* **24**, 2485 (2023). 36768808 10.3390/ijms24032485 PMC9916468

[16] Puchtler, H., Sweat, F. & Levine, M. On the binding of congo red by amyloid. *J. Histochem. Cytochem.* **10**, 355–364 (1962).

[17] Schindelin, J. et al. Fiji: an open-source platform for biological-image analysis. *Nat. Methods* **9**, 676–682 (2012). 22743772 10.1038/nmeth.2019 PMC3855844

[18] Liu, X., Liu, Y. & Ji, S. Secretases related to amyloid precursor protein processing. *Membranes* **11**, 983 (2021). 34940484 10.3390/membranes11120983 PMC8706128

[19] Ozdogan, E. & Arikan, C. Liver fibrosis in children: a comprehensive review of mechanisms, diagnosis, and therapy. *Clin. Exp. Pediatr.* **66**, 110–124 (2023). 36550776 10.3345/cep.2022.00367 PMC9989719

[20] Schoen, B. T., Lee, H., Sullivan, K. & Ricketts, R. R. The Kasai portoenterostomy: when is it too late? *J. Pediatr. Surg.* **36**, 97–99 (2001). 11150445 10.1053/jpsu.2001.20020

[21] Davenport, M., Gonde, C., Narayanaswamy, B., Mieli-Vergani, G. & Tredger, J. M. Soluble adhesion molecule profiling in preoperative infants with biliary atresia. *J. Pediatr. Surg.* **40**, 1464–1469 (2005). 16150350 10.1016/j.jpedsurg.2005.05.050

[22] Langenburg, S. E., Poulik, J., Goretsky, M., Klein, A. A. & Klein, M. D. Bile duct size does not predict success of portoenterostomy for biliary atresia. *J. Pediatr. Surg.* **35**, 1006–1007 (2000). 10873055 10.1053/jpsu.2000.6954

[23] Weerasooriya, V. S., White, F. V. & Shepherd, R. W. Hepatic fibrosis and survival in biliary atresia. *J. Pediatr.* **144**, 123–125 (2004). 14722530 10.1016/j.jpeds.2003.09.042

[24] Wu, L. N., Zhu, Z. J. & Sun, L. Y. Genetic factors and their role in the pathogenesis of biliary atresia. *Front. Pediatr.* **10**, 912154 (2022). 35844731 10.3389/fped.2022.912154 PMC9277099

[25] Stone, R. C. et al. Epithelial-mesenchymal transition in tissue repair and fibrosis. *Cell Tissue Res.* **365**, 495–506 (2016). 27461257 10.1007/s00441-016-2464-0 PMC5011038

[26] Venkat, V. et al. Childhood Liver Disease Research Network. Modeling outcomes in children with biliary atresia with native liver after 2 years of age. *Hepatol. Commun.* **4**, 1824–1834 (2020). 33305153 10.1002/hep4.1602 PMC7706301

[27] Gunadi et al. Collagen gene cluster expression and liver fibrogenesis in patients with biliary atresia: a preliminary study. *BMC Res. Notes* **16**, 356 (2023). 38041174 10.1186/s13104-023-06636-0 PMC10690962

[28] Kerola, A. et al. Molecular signature of active fibrogenesis prevails in biliary atresia after successful portoenterostomy. *Surgery* **162**, 548–556 (2017). 28655415 10.1016/j.surg.2017.04.013

[29] Jonigk, D. et al. Comparative analysis of morphological and molecular motifs in bronchiolitis obliterans and alveolar fibroelastosis after lung and stem cell transplantation. *J. Pathol. Clin. Res.* **3**, 17–28 (2016). 28138398 10.1002/cjp2.60 PMC5259562

[30] Madadi-Sanjani, O. et al. Growth factors assessed during Kasai procedure in liver and serum are not predictive for the postoperative liver deterioration in infants with biliary atresia. *J. Clin. Med.* **10**, 1978 (2021). 34062967 10.3390/jcm10091978 PMC8124311

[31] Anvarian, Z., Mykytyn, K., Mukhopadhyay, S., Pedersen, L. B. & Christensen, S. T. Cellular signalling by primary cilia in development, organ function and disease. *Nat. Rev. Nephrol.* **15**, 199–219 (2019). 30733609 10.1038/s41581-019-0116-9 PMC6426138

[32] Tam, P. K. H., Yiu, R. S., Lendahl, U. & Andersson, E. R. Cholangiopathies - towards a molecular understanding. *EBioMedicine* **35**, 381–393 (2018). 30236451 10.1016/j.ebiom.2018.08.024 PMC6161480

[33] McGaughran, J. M., Donnai, D. & Clayton-Smith, J. Biliary atresia in Kabuki syndrome. *Am. J. Med Genet.* **91**, 157–158 (2000). 10748418

[34] Lam, W. Y. et al. Identification of a wide spectrum of ciliary gene mutations in nonsyndromic biliary atresia patients implicates ciliary dysfunction as a novel disease mechanism. *EBioMedicine* **71**, 103530 (2021). 34455394 10.1016/j.ebiom.2021.103530 PMC8403738

[35] Davey, M. G., McTeir, L., Barrie, A. M., Freem, L. J. & Stephen, L. A. Loss of cilia causes embryonic lung hypoplasia, liver fibrosis, and cholestasis in the talpid3 ciliopathy mutant. *Organogenesis* **10**, 177–185 (2014). 24743779 10.4161/org.28819 PMC4154951

[36] Shen, W., Chen, G., Dong, R., Zhao, R. & Zheng, S. MicroRNA-21/PTEN/Akt axis in the fibrogenesis of biliary atresia. *J. Pediatr. Surg.* **49**, 1738–1741 (2014). 25487473 10.1016/j.jpedsurg.2014.09.009

[37] Babu, R. O. et al. Beta-amyloid deposition around hepatic bile ducts is a novel pathobiological and diagnostic feature of biliary atresia. *J. Hepatol.* **73**, 1391–1403 (2020). 32553668 10.1016/j.jhep.2020.06.012

[38] Tian, X. et al. amyloid deposition in biliary atresia reduces liver regeneration by inhibiting energy metabolism and mammalian target of rapamycin signaling. *Clin. Transl. Gastroenterol.* **13**, e00536 (2022). 36137184 10.14309/ctg.0000000000000536 PMC10476755

[39] Nagi, S. A. et al. Does amyloid b precursor protein gene expression have a role in diagnosis of biliary atresia? *Clin. Exp. Hepatol.* **9**, 335–345 (2023). 38774198 10.5114/ceh.2023.132818 PMC11103800

[40] Chou, C. W., Huang, Y. K., Kuo, T. T., Liu, J. P. & Sher, Y. P. An overview of ADAM9: structure, activation, and regulation in human diseases. *Int J. Mol. Sci.* **21**, 7790 (2020). 33096780 10.3390/ijms21207790 PMC7590139

[41] Bormann, T. et al. Role of matrix metalloprotease-2 and MMP-9 in experimental lung fibrosis in mice. *Respir. Res.* **23**, 180 (2022). 35804363 10.1186/s12931-022-02105-7 PMC9270768

[42] Giebeler, N. & Zigrino, P. A disintegrin and metalloprotease (ADAM): historical overview of their functions. *Toxins* **8**, 122 (2016). 27120619 10.3390/toxins8040122 PMC4848645

[43] Asai, M. et al. Putative function of ADAM9, ADAM10, and ADAM17 as APP alpha-secretase. *Biochem. Biophys. Res. Commun.* **301**, 231–235 (2003). 12535668 10.1016/s0006-291x(02)02999-6

[44] Perampalam, P. et al. Disrupting the DREAM transcriptional repressor complex induces apolipoprotein overexpression and systemic amyloidosis in mice. *J. Clin. Invest.* **131**, e140903 (2021). 33444292 10.1172/JCI140903 PMC7880409

[45] Yang, M. et al. Apolipoprotein A-II induces acute-phase response associated AA amyloidosis in mice through conformational changes of plasma lipoprotein structure. *Sci. Rep.* **8**, 5620 (2018). 29618729 10.1038/s41598-018-23755-y PMC5884826

[46] Löffek, S., Schilling, O. & Franzke, C. W. Series “matrix metalloproteinases in lung health and disease”: biological role of matrix metalloproteinases: a critical balance. *Eur. Respir. J.* **38**, 191–208 (2011). 21177845 10.1183/09031936.00146510

[47] Nomden, M., Beljaars, L., Verkade, H. J., Hulscher, J. B. F. & Olinga, P. Current concepts of biliary atresia and matrix metalloproteinase-7: a review of literature. *Front. Med.* **7**, 617261 (2020). 10.3389/fmed.2020.617261 PMC7779410 33409288

[48] Yang, L. et al. Diagnostic accuracy of serum matrix metalloproteinase-7 for biliary atresia. *Hepatology* **68**, 2069–2077 (2018). 30153340 10.1002/hep.30234 PMC6519383

[49] Aldeiri, B. et al. Matrix metalloproteinase-7 and osteopontin serum levels as biomarkers for biliary atresia. *J. Pediatr. Gastroenterol. Nutr.* **77**, 97–102 (2023). 37326848 10.1097/MPG.0000000000003792

[50] Zhou, X. et al. Expression of matrix metalloproteinase-2 and -14 persists during early resolution of experimental liver fibrosis and might contribute to fibrolysis. *Liver Int.* **24**, 492–501 (2004). 15482348 10.1111/j.1478-3231.2004.0946.x

[51] Otani, S. et al. Matrix metalloproteinase-14 mediates formation of bile ducts and hepatic maturation of fetal hepatic progenitor cells. *Biochem. Biophys. Res. Commun.* **469**, 1062–1068 (2016). 26724533 10.1016/j.bbrc.2015.12.105

[52] Jia, W., Rajani, C., Kaddurah-Daouk, R. & Li, H. Expert insights: the potential role of the gut microbiome-bile acid-brain axis in the development and progression of Alzheimer’s disease and hepatic encephalopathy. *Med. Res. Rev.* **40**, 1496–1507 (2020). 31808182 10.1002/med.21653

[53] Asefa, N. G. et al. Bioinformatic prioritization and functional annotation of GWAS-based candidate genes for primary open-angle glaucoma. *Genes* **13**, 1055 (2022). 35741817 10.3390/genes13061055 PMC9222386

[54] Coronel, R. et al. Amyloid precursor protein (APP) regulates gliogenesis and neurogenesis of human neural stem cells by several signaling pathways. *Int. J. Mol. Sci.* **24**, 12964 (2023). 37629148 10.3390/ijms241612964 PMC10455174

[55] Tang, X. et al. Transcriptomic and glycomic analyses highlight pathway-specific glycosylation alterations unique to Alzheimer’s disease. *Sci. Rep.* **13**, 7816 (2023). 37188790 10.1038/s41598-023-34787-4 PMC10185676

