# Supplementary material for: Amyloid precursor protein as a fibrosis marker in infants with biliary atresia
Source: Pediatr Res. 2024 Sep 28;97(5):1696–705. doi: 10.1038/s41390-024-03582-w (PMC12119347; doi:10.1038/s41390-024-03582-w)
Supplement: Supplementary file 1 — Supplementary Figure S1 [file 41390_2024_3582_MOESM1_ESM.pdf]

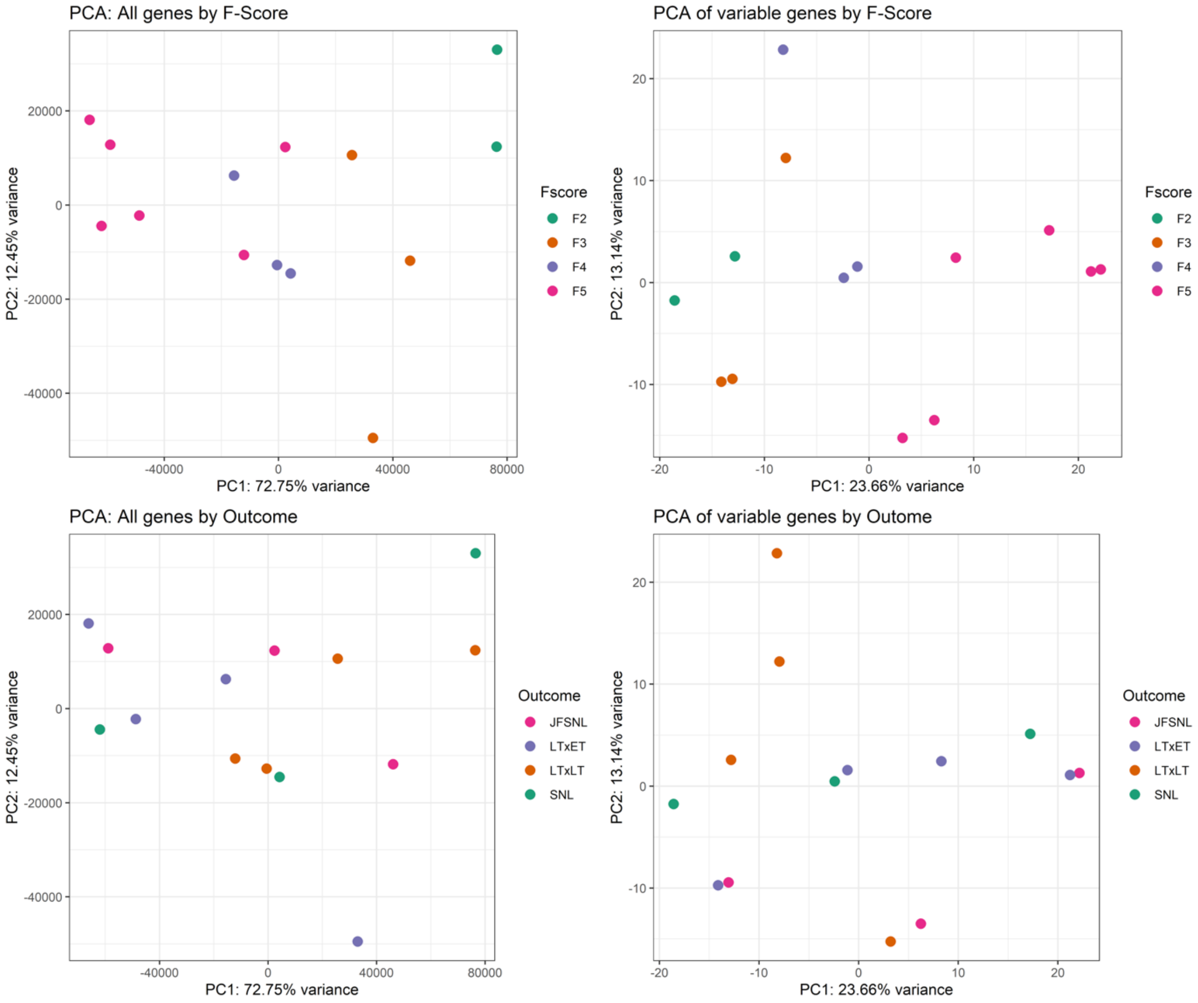


**Figure S1.** Principal component analysis (PCA) showed an excellent clustering of groups with different fibrosis levels (F2, F3, F4, F5), while different clinical outcome groups (jaundice-free and survival with native liver, JFSNL; lung transplant at early time point, LTxET; lung transplant at late time point, LTxLT; survival with native liver, SNL) did not show any perceptible clustering.
