# Supplementary material for: Amyloid precursor protein as a fibrosis marker in infants with biliary atresia
Source: Pediatr Res. 2024 Sep 28;97(5):1696–705. doi: 10.1038/s41390-024-03582-w (PMC12119347; doi:10.1038/s41390-024-03582-w)
Supplement: Supplementary file 2 — Supplementary Figure S2 [file 41390_2024_3582_MOESM2_ESM.pdf]

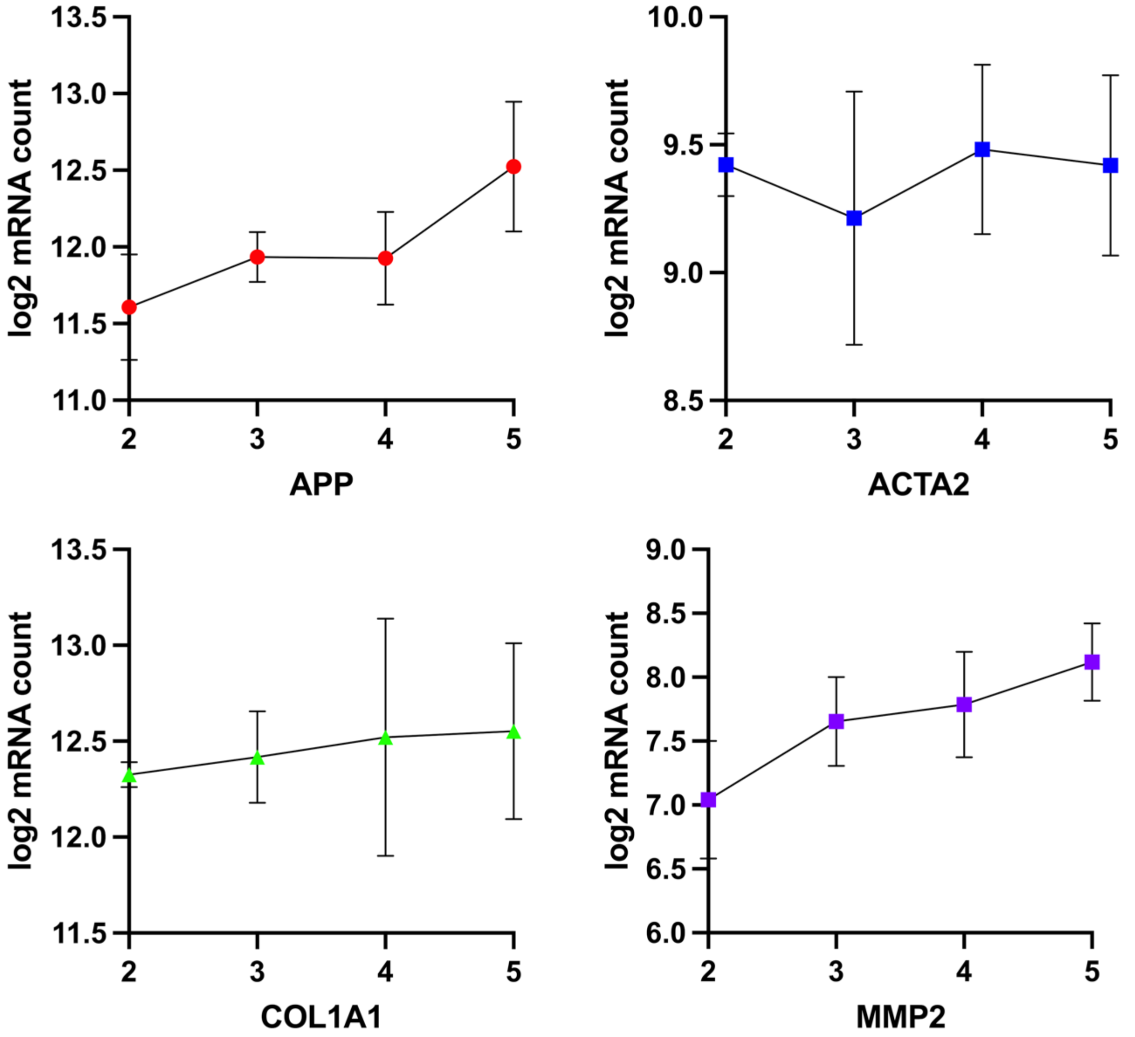

Figure S2. APP mRNA expression levels in comparison to well established fibrosis markers. Amyloid precursor protein, APP; alpha smooth muscle actin, ACTA2; matrix metalloproteinase 2, MMP2; collagen type 1A1, COL1A1.
