# Supplementary material for: Amyloid precursor protein as a fibrosis marker in infants with biliary atresia
Source: Pediatr Res. 2024 Sep 28;97(5):1696–705. doi: 10.1038/s41390-024-03582-w (PMC12119347; doi:10.1038/s41390-024-03582-w)
Supplement: Supplementary file 3 — Supplementary Table S1 [file 41390_2024_3582_MOESM3_ESM.pdf]

Table S1. Clinical characteristics

Patient	Diagnosis	Sex	Age at KPE / sampling (d)	Weight at KPE (g)	Clinical outcome	Syndromic BA variant	Ishak fibrosis score	Used for
01	BA	M	37	4680	LTx_LT	Yes	F4	mRNA, PCR, IHC
02	BA	F	42	3720	LTx_LT	No	F2	mRNA, PCR, IHC
03	BA	F	42	4820	LTx_LT	No	F3	mRNA, PCR, IHC, ELISA
04	BA	F	51	3700	JF_SNL	No	F5	mRNA, PCR, IHC, ELISA
05	BA	M	52	4200	LTx_ET	Yes	F5	mRNA, PCR, IHC
06	BA	F	52	4200	SNL	Yes	F2	mRNA, PCR, IHC
07	BA	F	54	4230	LTx_LT	No	F5	mRNA, PCR, IHC, ELISA
08	BA	M	54	4650	SNL	No	F5	mRNA, PCR, IHC
09	BA	M	58	4480	SNL	No	F4	mRNA, PCR, IHC
10	BA	M	73	6120	LTx_ET	No	F5	mRNA, PCR, IHC
11	BA	F	85	4800	JF_SNL	No	F3	mRNA, PCR, IHC
12	BA	M	86	6260	JF_SNL	No	F5	mRNA, PCR, IHC
13	BA	M	92	4440	LTx_ET	No	F3	mRNA, PCR, IHC
14	BA	F	110	6000	LTx_ET	No	F4	mRNA, PCR, IHC
15	BA	F	59	4600	LTx_ET	no	F5	ELISA
16	BA	M	28	4414	LTx_ET	no	F3	ELISA
17	BA	F	43	4370	SNL	no	n/a	ELISA
18	BA	F	26	3900	JF_SNL	no	F4	ELISA
19	BA	F	68	4350	JF_SNL	no	n/a	ELISA
20	BA	M	78	5760	JF_SNL	no	F3	ELISA
21	BA	F	94	5310	SNL	yes	F3	ELISA
22	BA	F	59	5640	n/a	no	n/a	ELISA
23	BA	F	41	4292	n/a	no	n/a	ELISA
24	BA	F	74	5335	LTx_ET	no	n/a	ELISA
25	BA	F	17	2300	LTx_ET	yes	F3	ELISA
26	BA	F	49	5370	JF_SNL	no	F4	ELISA
27	BA	M	49	3020	SNL	yes	n/a	ELISA
28	BA	F	45	3134	n/a	no	F2	ELISA
29	BA	M	23	4860	JF_SNL	no	F4	ELISA
30	BA	M	41	4460	LTx_ET	no	F3	ELISA
31	BA	M	75	4620	LTx_ET	no	n/a	ELISA
32	BA	M	45	5910	SNL	no	n/a	ELISA
33	BA	F	34	4130	SNL	no	n/a	ELISA
34	BA	F	66	4240	LTx_ET	no	F5	ELISA
35	BA	M	51	4050	n/a	no	F5	ELISA
36	BA	F	51	4212	LTx_ET	no	F4	ELISA
37	BA	F	76	3940	Death	no	F5	ELISA
38	BA	M	64	5550	SNL	no	F4	ELISA
39	BA	F	59	3875	LTx_ET	no	n/a	ELISA
40	BA	M	46	4320	JF_SNL	no	F4	ELISA
41	BA	M	30	3800	JF_SNL	no	F4	ELISA
42	BA	M	90	5470	JF_SNL	no	n/a	ELISA
43	BA	M	47	4800	LTx_ET	no	F3	ELISA
44	BA	F	22	3895	LTx_ET	yes	F4	ELISA
45	IH	M	32	4700				ELISA
46	IH	M	66	3000				ELISA
47	IH	M	93	4000				ELISA
48	IH	M	46	4122				ELISA
49	IH	M	104	5080				ELISA
50	IH	M	42	4500				ELISA
51	IH	F	51	2700				ELISA
52	IH	M	62	5800				ELISA
53	IH	M	56	4165				ELISA
54	IH	M	56	3675				ELISA
55	Healthy	M	38 yrs	n/a				IHC
56	Healthy	W	32 yrs	n/a				IHC
57	Healthy	W	41 yrs	n/a				IHC
58	Healthy	W	54 yrs	n/a				IHC
59	Healthy	W	44 yrs	n/a				IHC
60	Healthy	n/a	n/a	n/a				IHC

KPE, Kasai portenterostomy; BA, biliary atresia; IH, inguinal hernia; mRNA, messenger ribonucleic acid; PCR, polymerase chain reaction; IHC, immunohistochemistry; ELISA, enzyme-linked immunosorbent assay; n/a, not available; M, male; F, female; d, days; g, gramm; yrs, years.
